# Notes and News

**Published:** 1905-01-14

**Authors:** 


### NOTES AND NEWS.

THE new Hospital for Consumption at Liverpool will accommodate 30 patients. Verandahs are provided on one side of the building, and the flat roofs will be utilised as "airing grounds" for the patients.

SIR EDGAR VINCENT has promised £100 to meet a promise of a resident of Esher who has offered a like sum to the Royal Waterloo Hospital for Children and Women, provided that four other donors come forward.

A NEW isolation hospital has been opened at Morton. The cost has amounted to £6,857, which is smaller than the sum originally estimated; a very unusual fact in regard to estimates, which is worthy of notice in consequence.

IT is proposed to establish a West Wales Consumption Sanatorium at Llanyfyther. It is understood that the employees at the Pembroke Dockyard will support two beds, and efforts are being made in other directions to secure means of carrying on the work. The project provides for 20 beds, and Princess Christian has consented to lay the foundation stone of the building.

WE regret to record the death of Dr. Murray Braidwood, who was a well-known member of the medical profession. He took his M.D. at Edinburgh in 1863, and became F.R.C.S.Edin. in 1880. He studied at Berlin, Prague, and Vienna. He gained the Fotheringham Medal, the Boyston Prize (Havard University), and his thesis for the M.D. degree gained him the gold medal. He was examiner in medical jurisprudence at the Edinburgh University, and served on the medical staff at the Edinburgh Royal Infirmary, Cumberland Infirmary, and Worral Hospital for Sick Children. He made some useful contributions to medical literature.

SCARLET fever has broken out in the Burton-on-Trent Poor-law Hospital, where there are also cases of measles. The resources of the hospital are quite unfitted to cope with the outbreak.

THE Hospital Saturday Fund have decided to contribute £500 to the National Sanatorium Committee towards the Sanatorium for Consumption to be erected near Hastings.

THE St. Helens Hospital has been extended and can now accommodate twice as many patients as formerly. The legacy of the late Miss Gorton, of Southport, will be devoted to the endowment of the new buildings.

THE King and Queen visited the Devonshire Hospital, Buxton, on Saturday, and the King offered to present a new operating-table to the institution. The Royal visitors expressed themselves pleased with the arrangements for the comfort of the patients.

THE annual contributions sent to the King's Hospital Fund by the Prince of Wales and his family makes an interesting example for family contributions by the King's subjects. The Prince of Wales heads the list with a donation of £300. The Princess follows with one of £52 10s., and the four little Princes and Princess Victoria each present £1 to the hospitals.

THE secretary-superintendent of the Cardiff Infirmary makes an interesting New Year appeal on behalf of the institution. He asks for charity—not as expressed by the giving of funds, but as expressed by kindliness of thought and action in regard to the hospital, so that it may not suffer from detractors and malcontents. He asks that all causes of dissatisfaction may, in the first instance, be referred to him, that an opportunity may be given to rectify any cause for dissatisfaction before it is discussed elsewhere to the detriment of the institution. Sir William Thomas Lewis has given £1,000 to the Infirmary.

IT may be remembered that the extensive scheme to inoculate some millions of the inhabitants of India with Haffkine plague serum received a check only a few months after its operations were commenced. This was owing to an alarming outbreak of tetanus, which was traced to the use of the serum. Investigations followed, which showed that, in order to expedite the production of the serum, the use of carbolic in its preparation had been omitted. A consensus of opinion declared the omission to be a grave mistake, an opinion which has been endorsed by the Lister Institute. Consequently the carbolic is to be restored to the preparation, and, in order to restore public confidence, a series of public demonstrations in the process of manufacture were arranged to take place. These are being held by the Director of the Plague Research Laboratory in connection with the Health Exhibition now being held in Bombay under the direction of the Bombay Sanitary Association.

